# Peripheral adaptive immune remodeling associated with severe hearing loss in older adults: a single-center flow-cytometry study with mouse cochlear transcriptomic context

**DOI:** 10.3389/fragi.2026.1925166

**Published:** 2026-09-03

**Authors:** Qunhui Zhang, Wei Liu, Meijun Shao, Jinyu Wang, Qianyi Zhong, Linlan Jiang

**Affiliations:** Guangzhou Twelfth People’s Hospital, Guangzhou, Guangdong, China

**Keywords:** adaptive immune remodeling, flow cytometry, mouse cochlea, older adults, severe hearing loss, transcriptomic context

## Abstract

**Objective:**

To investigate the association between severe hearing loss and systemic immune aging in older adults using participant-level clinical immunophenotyping, and to explore potential inflammatory pathways and cellular contexts using public mouse cochlear transcriptomic data.

**Methods:**

We analyzed a single-center cohort of 76 older adults, classified into normal hearing (NH), mild-to-moderate hearing loss (MMHL), or severe-to-profound hearing loss (SPHL) groups. Age- and sex-adjusted models were used to assess immune profiles. Prespecified sensitivity and diagnostic analyses evaluated age specification, overlap weighting, influential observations, and the stability of the inversion model. Additionally, public mouse cochlear bulk transcriptomics and single-nucleus RNA sequencing (snRNA-seq) data were examined to identify relevant inflammatory pathways and cell-type-specific immune-stress modules.

**Results:**

SPHL was significantly associated with a higher T-cell/CD8 skew, increased CD4 differentiation, an elevated EM/naive CD4 balance, a lower CD8 activation/checkpoint score, and increased odds of CD4/CD8 inversion. These associations remained consistent across sensitivity analyses. Mouse bulk transcriptomics highlighted inflammatory response and IL6/JAK/STAT3 signaling pathways, while snRNA-seq localized immune-stress modules primarily to broad macrophage and perivascular macrophage-like cell populations.

**Conclusion:**

Severe hearing loss in older adults is associated with distinct peripheral immune remodeling features indicative of systemic immune aging. These findings support an association-focused framework linking hearing loss severity with immune dysregulation, though validation in larger, clinically annotated cohorts is required.

## Introduction

Hearing loss in older adults is common, clinically heterogeneous, and often evaluated through audiometric severity (Gates and Mills, 2005; Lin et al., 2011; Yamasoba et al., 2013; Goman and Lin, 2016; Cunningham and Tucci, 2017; Bowl and Dawson, 2019; World Health Organization, 2021). Severe hearing loss is particularly important because it is more likely to affect communication, social functioning, and clinical decision-makin (Bainbridge and Wallhagen, 2014; Cunningham and Tucci, 2017). However, audiometric categories alone do not explain why some older adults show more severe loss than others, nor do they describe systemic biological states that may accompany severe hearing impairment. Immune aging is one plausible contributor to this broader biological context, but its relationship with hearing-severity status remains incompletely described in clinical cohorts (Franceschi et al., 2000; Fulop et al., 2017; Nikolich-Žugich, 2018; Pawelec, 2018; Parekh and Kaur, 2023).

Peripheral immune aging is characterized by redistribution across adaptive immune compartments, including changes in CD4 and CD8 T-cell balance, naive and effector-memory states, activation markers, checkpoint-associated phenotypes, and inversion of the CD4/CD8 ratio (Wikby et al., 2005; Strindhall et al., 2013; Luz Correa et al., 2014; Nikolich-Žugich, 2018; Goronzy and Weyand, 2019). These features are measurable in peripheral blood by flow cytometry and can be summarized at both marker and composite-score levels. A blood immune phenotype cannot be assumed to represent cochlear tissue biology, but it can provide a participant-level readout for testing whether severe hearing loss is accompanied by systemic adaptive immune remodeling.

Human cochlear tissue is rarely available from clinical hearing-loss cohorts, and age-related cochlear pathology is biologically heterogeneous (Yamasoba et al., 2013; Keithley, 2020; Parekh and Kaur, 2023). Public mouse cochlear transcriptomic datasets therefore offer a limited but useful reference frame. Bulk cochlear data can identify pathway-level inflammatory signals in tissue, while single-cell or single-nucleus data can show which broad cochlear cell-label groups carry selected immune or stress modules (Frye et al., 2017; Seicol et al., 2022; Xia et al., 2025). These datasets do not validate the human peripheral blood findings, and they cannot establish a paired blood-to-cochlea mechanism. Their value in the present study is narrower: they provide tissue-contextual information that helps frame, but not prove, the biological plausibility of the clinical associations.

We therefore designed the analysis around a predefined evidence hierarchy. The clinical flow-cytometry cohort was used for participant-level association analyses. Public mouse cochlear bulk transcriptomics was used for pathway context. Public mouse cochlear snRNA-seq was used for broad-label localization context among assigned cells. Throughout the manuscript, these layers are kept separate to avoid implying causality, immune infiltration, diagnostic prediction, or human cochlear validation. Public mouse cochlear datasets provide contextual support, but do not validate the human findings.

## Materials and methods

### Study design and evidence hierarchy

This was an association-focused, single-center clinical immunophenotyping study with public mouse cochlear transcriptomic context. The clinical dataset was used to test whether SPHL was associated with predefined peripheral immune outcomes after adjustment for age and sex. The mouse datasets were not paired with the clinical cohort, did not include human cochlear measurements from the same participants, and were analyzed as contextual resources only.

The evidence hierarchy was defined before manuscript assembly. First, the participant-level clinical layer provided the main inferential evidence. Second, GSE49543 provided mouse cochlear bulk pathway context for inflammatory and stress-related programs. Third, GSE274279 provided mouse cochlear snRNA-seq broad-label localization context. This organization was used in the figures, tables, and interpretation so that tissue-context layers were not presented as validation of the clinical blood data.

### Clinical cohort and hearing definition

The analyzed cohort included 76 older adult participants recruited from the Department of Otolaryngology, Guangzhou Twelfth People’s Hospital, between April 2024 and June 2025. The cohort included 21 participants with normal hearing (NH), 32 with mild-to-moderate hearing loss (MMHL), and 23 with severe-to-profound hearing loss (SPHL). The primary clinical contrast was SPHL versus non-SPHL, where non-SPHL comprised the NH and MMHL groups (n = 53). The cohort age range was 56–95 years; the SPHL age-overlap sensitivity subset covered 61–88 years. Participants with conditions known to significantly affect immune status were excluded. Specifically, we excluded individuals with recent acute infections (within 4 weeks), active malignancies, a history of autoimmune diseases, or recent vaccination (within 4 weeks). Participants who had used systemic antibiotics, corticosteroids, or other immunomodulatory drugs within the past 3 months were also excluded.

Hearing-severity groups were defined by bilateral average threshold across 125, 250, 500, 1000, 2000, 4000, and 8000 Hz, consistent with the broader use of pure-tone thresholds for hearing-loss classification while recognizing that frequency selection and better-ear versus bilateral definitions vary across epidemiologic studies (Cruickshanks et al., 1998; Agrawal et al., 2008; Goman and Lin, 2016; World Health Organization, 2021). NH was defined as <= 25 dB HL, MMHL as 26–60 dB HL, and SPHL as >60 dB HL. Individual threshold values and frequency-specific thresholds were not available in the clinical analysis dataset; therefore, continuous-threshold models, frequency-specific models, and alternative PTA definitions were not performed. This limitation is important because hearing severity, age, and systemic immune aging are biologically and clinically related.

### Flow cytometry and derived immune outcomes

Peripheral blood flow-cytometry acquisition and quality control followed the laboratory’s routine standard operating procedures, and reporting was organized with reference to established flow-cytometry reporting and interpretation principles (Perfetto et al., 2004; Herzenberg et al., 2006; Lee et al., 2008; Cossarizza et al., 2021). The antibody panel, representative available gating hierarchy, parent populations, and derived score definitions are summarized in Supplementary Tables S1-S3 and Supplementary Figure S1. The representative gating figure is methodology evidence only and is not used as group-comparison data. Peripheral blood was collected in heparin sodium tubes and processed within 4 h. Fluorescence Minus One (FMO) controls were used. A minimum of 50,000 T-cell events were collected.

The main immune outcomes were predefined from the clinical analysis outputs and included T-cell/CD8 skew, CD4 differentiation, log EM/naive CD4, CD8 activation/checkpoint score, NK natural-cytotoxicity receptor (NCR) score, and CD4/CD8 inversion. Composite score components and directionality are listed in Supplementary Table S3. CD4/CD8 inversion was treated as a binary event and analyzed separately from continuous immune outcomes.

To characterize overlap among the six prespecified immune outcomes, we calculated all 15 unique two-sided Spearman correlations. Every pair included the full cohort (n = 76), and Benjamini–Hochberg correction was applied across the 15 correlation tests. Correlations involving CD4/CD8 inversion were interpreted descriptively because inversion was a binary outcome. The correlation matrix and complete statistics are reported in Supplementary Figure S3 and Supplementary Data S4, respectively.

### Clinical association models

The primary exposure was SPHL versus non-SPHL. Continuous immune outcomes were modeled with age- and sex-adjusted linear models using HC3 robust standard errors (MacKinnon and White, 1985). CD4/CD8 inversion was modeled as a binary outcome and reported as an odds ratio with 95% confidence interval. P values for the primary exposure terms were interpreted alongside false-discovery rate (FDR) adjustment within the prespecified clinical outcome set (Benjamini and Hochberg, 1995).

Sensitivity analyses were used to assess whether the main direction of association was stable under alternative analytic summaries. Age-overlap restricted analysis used the overlapping age range between SPHL and non-SPHL groups (61–88 years), retaining 73 participants (SPHL n = 23, non-SPHL n = 50). Weighted sensitivity and ordered-severity sensitivity outputs were summarized as robustness checks. These analyses were not treated as independent replication and do not remove the possibility of residual confounding.

No unavailable clinical covariates were imputed. Variables such as individual audiometric thresholds, frequency-specific thresholds, comorbidities, medication history, exposure history, inflammatory markers, and infection-related markers were not available in the clinical analysis dataset. Consequently, the models should be interpreted as adjusted associations rather than causal estimates.

Model assessment included residual and influence summaries for the five continuous outcomes, nonlinear-age models using a natural cubic spline with 3 degrees of freedom, leave-one-out refitting of the continuous models, and covariate-balance assessment after overlap weighting. Stability of the CD4/CD8 inversion model was examined with a stratified participant-level nonparametric bootstrap of 2,000 replicates, with the mean bias-reduced model refitted in each replicate. Detailed model specifications, diagnostics, weighting balance, bootstrap results, and the unified six-outcome FDR calculation are provided in Supplementary Table S4B.

### Public mouse cochlear bulk transcriptomic context

GSE49543 was summarized as a public mouse cochlear bulk transcriptomic context dataset (NCBI GEO, GSE49543). The curated primary contrast compared reference mouse cochlea samples (n = 9) with severe mouse cochlea samples (n = 6). Figure 4 presents sample-level PCA context, gene-level transparency, primary FDR-supported pathway anchors, and secondary exploratory pathway context. Positive normalized enrichment score (NES) indicates enrichment in severe mouse cochlea relative to reference in the curated contrast.

Gene-set enrichment was performed on the moderated t-statistic ranking using fgseaMultilevel with minimum and maximum gene-set sizes of 10 and 500, respectively. Hallmark and Reactome collections were obtained through msigdbr, and the prespecified immune-focused subset was evaluated separately (Subramanian et al., 2005; Liberzon et al., 2015; Ritchie et al., 2015). Complete Hallmark, Reactome, and immune-focused enrichment results, together with sample metadata and method provenance, are provided in Supplementary Data S1. The pathways emphasized in the main figure were selected from the prespecified immune-focused display; the complete enrichment output was retained in Supplementary Data S1.

### Public mouse cochlear snRNA-seq context

GSE274279 was summarized as public mouse cochlear snRNA-seq broad-label localization context ((Xia et al., 2025), NCBI GEO, GSM8446835). The final QC-filtered broad-label/module-scored universe included 11,143 cells, after reconciling the initial/pre-filter 11,485-cell object with 342 cells removed during QC filtering. Among the final universe, 3,837 cells were assigned to broad labels and 7,306 remained unresolved.

UMAP, broad-label composition, marker-evidence, and module-localization summaries were based on exported source tables, with interpretation informed by standard single-cell analysis and visualization practices (Hafemeister and Satija, 2019; Stuart et al., 2019; McInnes et al., 2020). The analysis did not infer cell abundance shifts. Unresolved cells were retained as QC/background and excluded from assigned-label ranking. Module-localization values were interpreted as row-scaled relative scores among assigned broad labels; a value of 1.00 indicates the highest-scoring assigned broad label within that module, not an absolute enrichment or cell-proportion estimate.

Supplementary Data S2 provides the exported sample structure, applied QC thresholds and retained counts, broad-label marker evidence, module gene sets, module-localization summaries, marker-overlap audit, and method provenance used for the reviewer-facing analysis.

## Results

### Study structure and evidence boundaries

**FIGURE 1:**
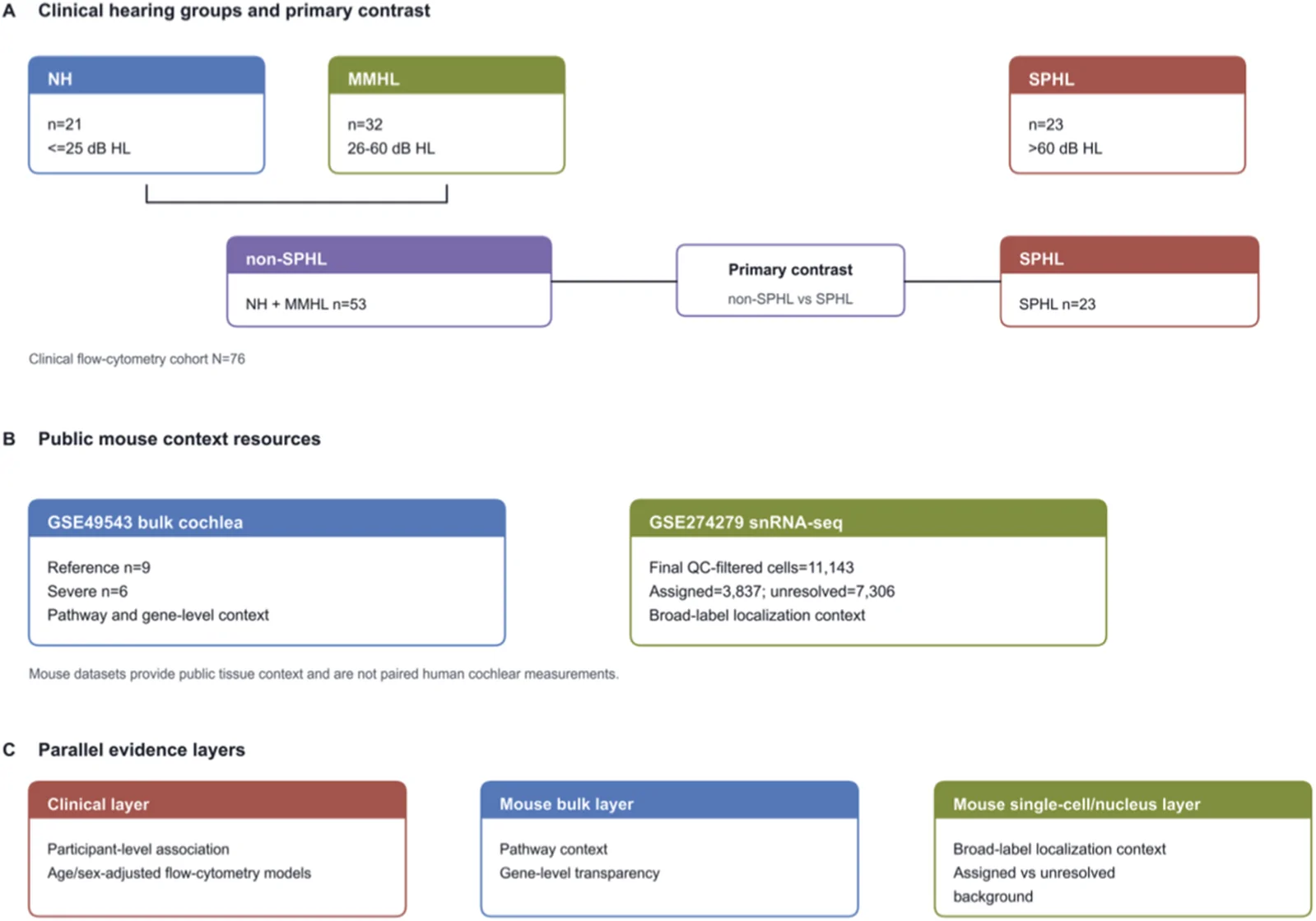
Study design and evidence framework summarize the clinical cohort, the primary SPHL versus non-SPHL contrast, and the two public mouse cochlear context resources. Panel **(A)** shows the hearing groups used for the clinical analysis: NH (n = 21), MMHL (n = 32), and SPHL (n = 23). NH and MMHL were combined as non-SPHL (n = 53) for the primary contrast against SPHL. Panel **(B)** summarizes the public mouse context datasets: GSE49543 bulk cochlea (reference n = 9, severe n = 6) and GSE274279 snRNA-seq broad-label context (11,143 final QC-filtered cells; 3,837 assigned; 7,306 unresolved). Panel **(C)** presents the clinical layer, mouse bulk layer, and mouse single-cell/nucleus layer as parallel evidence layers rather than a validation chain. The interpretation boundary is: association only; public mouse context only; no causality; no abundance/infiltration inference; no human cochlear validation. Cohort characteristics and descriptive immune landscape.

Figure 1 summarizes the study structure. The clinical cohort was the only participant-level inference layer and included 76 participants across three hearing-severity groups: NH (n = 21), MMHL (n = 32), and SPHL (n = 23). The primary contrast combined NH and MMHL as non-SPHL (n = 53) and compared this group with SPHL. This contrast was selected to focus the clinical analysis on severe-to-profound hearing loss rather than on all pairwise differences among hearing categories.

The two public mouse datasets were used as context resources and not as validation cohorts. GSE49543 contributed bulk cochlear pathway information, while GSE274279 contributed broad-label snRNA-seq localization information. Figure 1 summarizes the final GSE274279 context counts (11,143 final QC-filtered cells, 3,837 assigned cells, and 7,306 unresolved cells), while Figure 5 provides the full initial-to-final cell-count reconciliation from 11,485 initial/pre-filter cells to the final 11,143-cell universe.

### Cohort characteristics and descriptive immune landscape

Table 1 summarizes the primary SPHL versus non-SPHL contrast. The total cohort included 76 participants, with 53 in the non-SPHL group and 23 in the SPHL group. Median age was 71.0 years in the total cohort, 71.0 years in non-SPHL, and 72.0 years in SPHL. Female sex was recorded in 41 of 76 participants overall, 31 of 53 in non-SPHL, and 10 of 23 in SPHL. Table 1 is intentionally limited to variables available in the clinical analysis dataset.

**TABLE 1:** Cohort characteristics for the primary SPHL versus non-SPHL contras.

Variable	Total (n = 76)	Non-SPHL (n = 53)	SPHL (n = 23)	SMD (standardized mean difference)
Participants, n	76	53	23	—
Age range, years	56-95	56-95	61-88	—
Age, years, median [IQR]	71.0 [66.0, 77.2]	71.0 [65.0, 76.0]	72.0 [66.0, 78.5]	0.27
Female sex, n (%)	41 (53.9%)	31 (58.5%)	10 (43.5%)	−0.3

**FIGURE 2:**
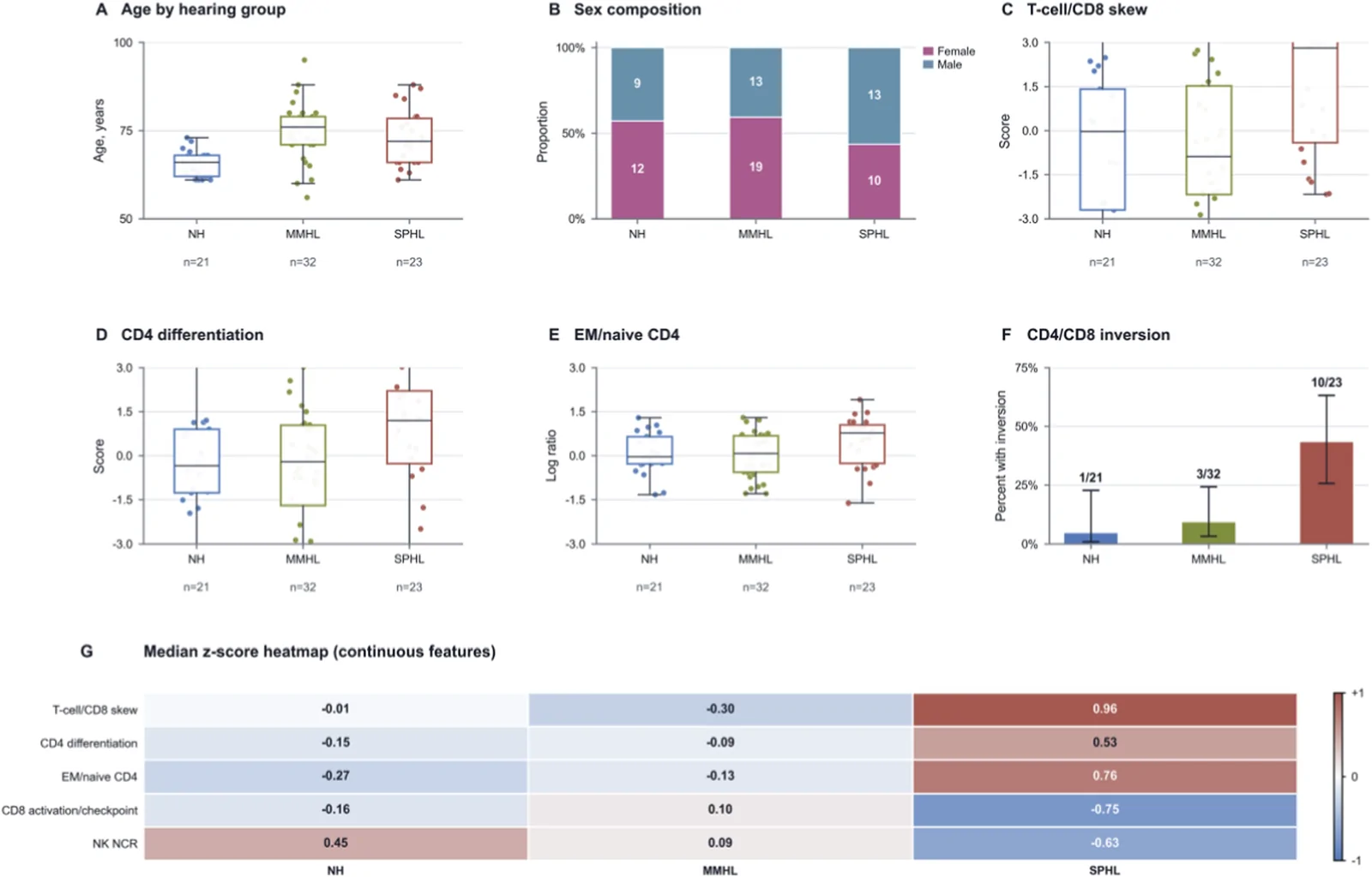
Descriptive clinical immune landscape displays cohort structure and selected immune-feature distributions by hearing group. The figure is descriptive and is not used as the primary inferential analysis; adjusted clinical inference is reported in Figure 3 and Table 2. Panel **(A)** shows age distributions, Panel **(B)** shows sex composition (pink denotes female participants and blue denotes male participants), and Panels **(C–E)** show participant-level distributions for selected continuous immune features. Panel **(F)** shows CD4/CD8 inversion as a binary event with group-specific event counts (NH 1/21, MMHL 3/32, SPHL 10/23). Panel **(G)** summarizes group median z-scores for selected continuous immune features. CD4/CD8 inversion is excluded from the median z-score heatmap because it is analyzed as a binary event.

Figure 2 displays age, sex composition, selected continuous immune features, CD4/CD8 inversion, and group median z-scores. The figure is descriptive and is not intended to replace adjusted clinical inference. CD4/CD8 inversion is shown as a binary event with event counts of 1/21 in NH, 3/32 in MMHL, and 10/23 in SPHL. Because CD4/CD8 inversion is a binary event, it is excluded from the median z-score heatmap.

### Adjusted clinical associations and robustness analyses

**FIGURE 3:**
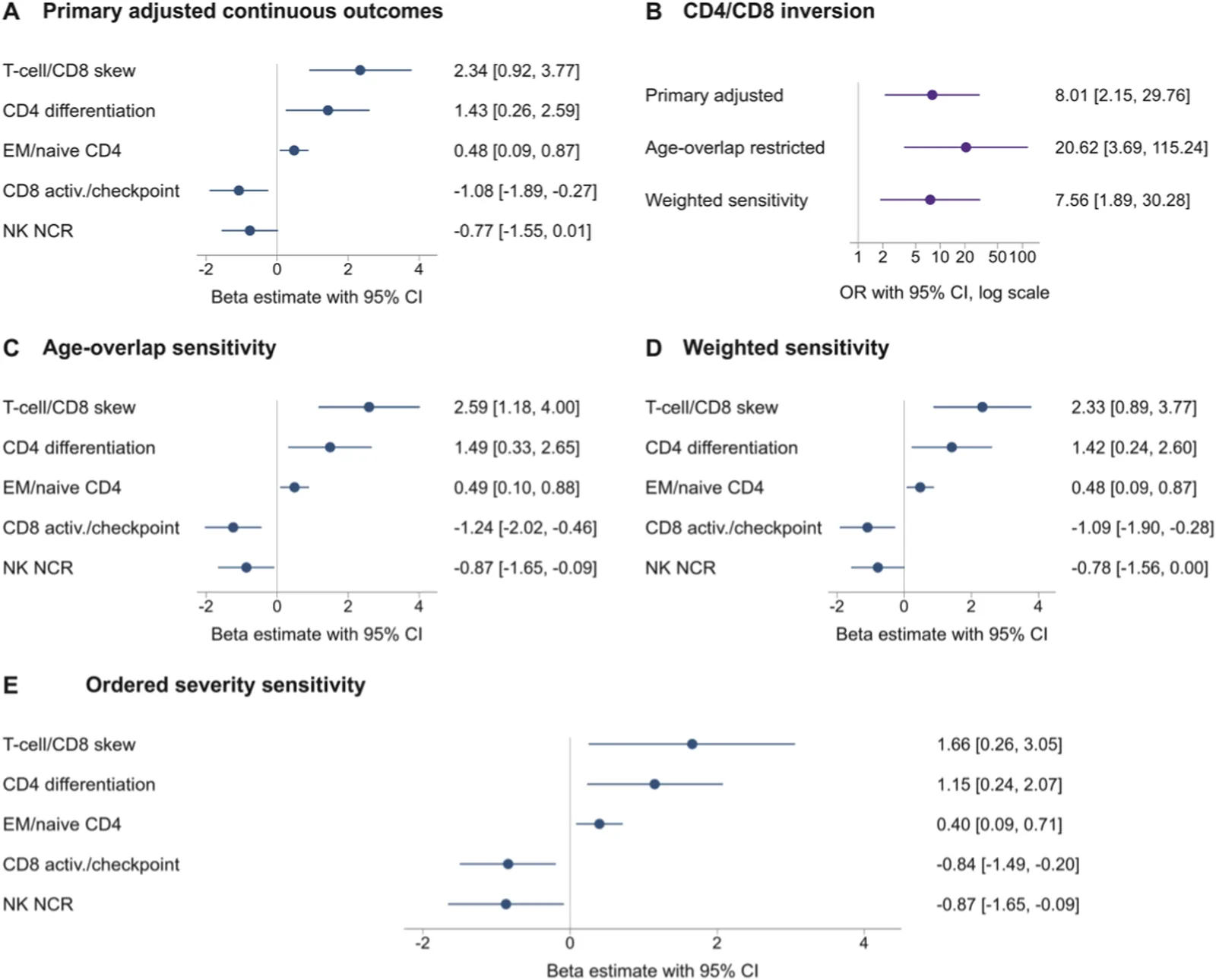
Clinical association and robustness summarize the clinical association models and sensitivity analyses for the single-center flow-cytometry cohort. Continuous outcomes were evaluated using age- and sex-adjusted linear models with HC3 robust standard errors and are shown as beta estimates with 95% confidence intervals. CD4/CD8 inversion is displayed separately as an odds ratio with 95% confidence interval on a log scale; event counts were non-SPHL 4/53 and SPHL 10/23. Panels **(A,C,D,E)** use the same outcome order and forest-plot template for continuous outcomes. Panel **(A)** shows the primary adjusted models, Panel **(B)** presents the primary adjusted, age-overlap restricted, and weighted sensitivity analyses for CD4/CD8 inversion, Panel **(C)** the age-overlap restricted sensitivity, Panel **(D)** the weighted sensitivity, and Panel **(E)** the ordered-severity sensitivity. Sensitivity analyses are robustness checks and do not constitute independent validation. Detailed model diagnostics, weighting balance, bootstrap results, and unified FDR values are provided in Supplementary Table S4B.

Figure 3 and Table 2 provide the main clinical association results. In primary age- and sex-adjusted models, SPHL was associated with higher T-cell/CD8 skew (beta 2.34, 95% CI 0.92–3.77), higher CD4 differentiation (beta 1.43, 95% CI 0.26–2.59), higher EM/naive CD4 (beta 0.48, 95% CI 0.09–0.87), and lower CD8 activation/checkpoint score (beta −1.08, 95% CI -1.89 to −0.27). NK NCR showed a directionally similar but weaker primary estimate (beta −0.77, 95% CI -1.55 to 0.01).

**TABLE 2:** Age- and sex-adjusted clinical models and sensitivity analyses.

Outcome	Primary estimate	95% CI	P Value	FDR q	Age-overlap estimate	Weighted estimate	Ordered-severity estimate
T-cell/CD8 skew	2.34	[0.92, 3.77]	0.0019	0.0096	2.59 [1.18, 4.00]	2.33 [0.89, 3.77]	1.66 [0.26, 3.05]
CD4 differentiation	1.43	[0.26, 2.59]	0.019	0.024	1.49 [0.33, 2.65]	1.42 [0.24, 2.60]	1.15 [0.24, 2.07]
EM/naive CD4	0.48	[0.09, 0.87]	0.019	0.024	0.49 [0.10, 0.88]	0.48 [0.09, 0.87]	0.40 [0.09, 0.71]
CD8 activation/checkpoint	−1.08	[-1.89, −0.27]	0.011	0.024	−1.24 [-2.02, −0.46]	−1.09 [-1.90, −0.28]	−0.84 [-1.49, −0.20]
NK NCR	−0.77	[-1.55, 0.01]	0.057	0.057	−0.87 [-1.65, −0.09]	−0.78 [-1.56, 0.00]	−0.87 [-1.65, −0.09]
CD4/CD8 inversion	8.01	[2.15, 29.76]	0.0019	0.0019	20.62 [3.69, 115.24]	7.56 [1.89, 30.28]	—

1 Continuous outcomes were modeled using age- and sex-adjusted linear models with HC3 robust standard errors.

2 CD4/CD8 inversion was analyzed as a binary outcome and reported as an odds ratio. CD4/CD8 inversion events: non-SPHL, 4/53; SPHL, 10/23.

3 Sensitivity analyses are robustness analyses and should not be interpreted as independent replication.

CD4/CD8 inversion was more frequent in SPHL, and was modeled separately as a binary event. The primary adjusted odds ratio for SPHL, versus non-SPHL, was 8.01 (95% CI, 2.15–29.76), with event counts of 10/23 in SPHL, and 4/53 in non-SPHL., The binary odds-ratio scale is therefore displayed separately from the continuous immune-score beta estimates.

The sensitivity and diagnostic analyses retained the primary effect directions. The five continuous outcomes retained their directions in the age-overlap, overlap-weighted, ordered-severity, and nonlinear-age analyses, and all 380 leave-one-out refits completed without a direction reversal. After overlap weighting, residual age and sex imbalance was negligible and the Kish effective sample size was 61.86. For CD4/CD8 inversion, all 2,000 bootstrap replicates were valid; the median bootstrap odds ratio was 9.74 and the percentile 95% interval was 2.47–86.08. Complete model diagnostics and sensitivity results are reported in Supplementary Table S4B.

The six prespecified outcomes showed a partially overlapping correlation structure (Supplementary Figure S3 and Supplementary Data S4). Seven of the 15 pairwise correlations remained significant after Benjamini–Hochberg correction. The strongest correlation was between CD4 differentiation and EM/naive CD4 (Spearman rho = 0.880, q < 0.001). These results indicate that the prespecified outcomes capture related, but not identical, dimensions of peripheral immune phenotype.

### Public mouse cochlear bulk pathway context

**FIGURE 4:**
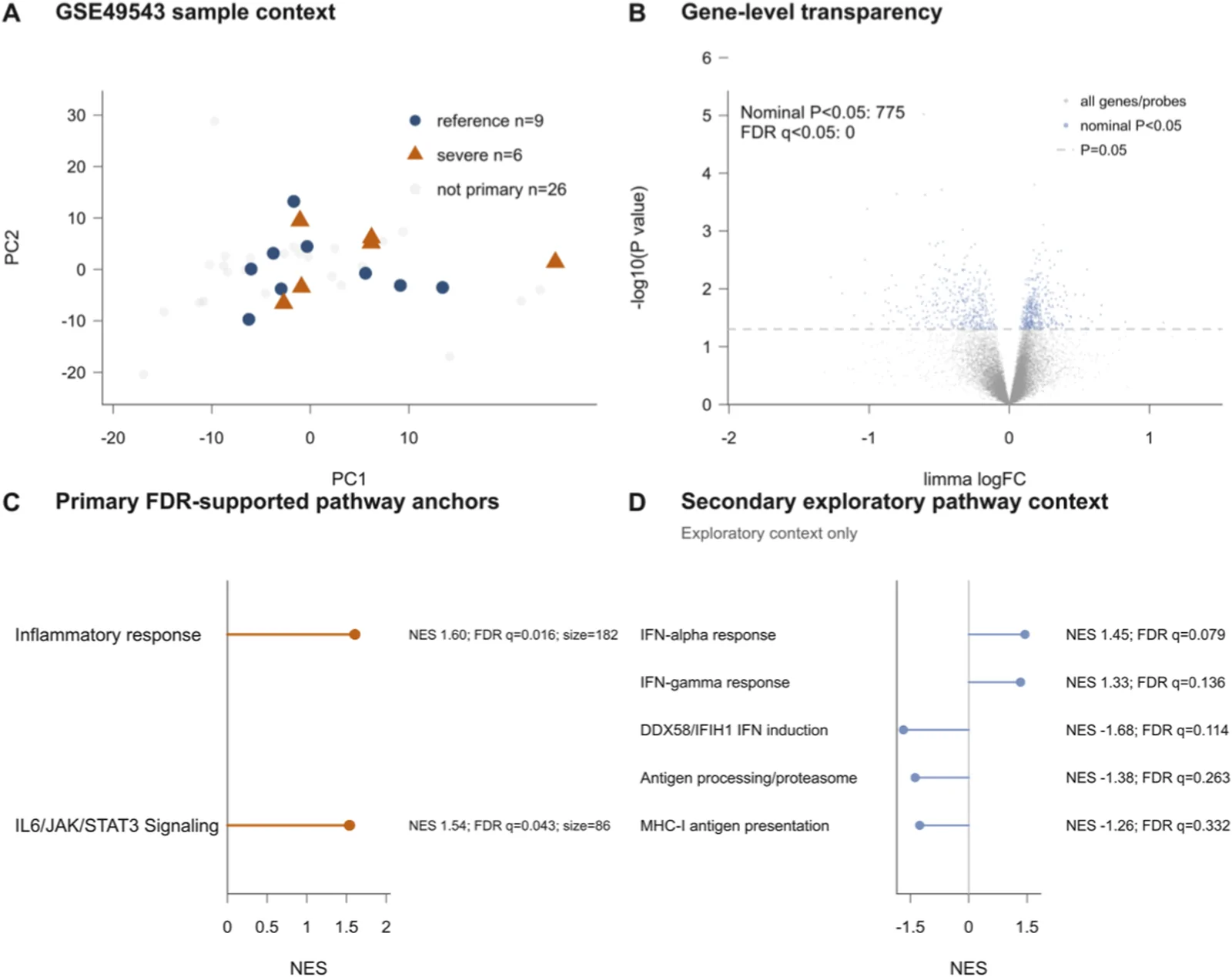
Public mouse cochlear bulk transcriptomic context for inflammatory pathway anchors summarizes GSE49543 public mouse cochlear bulk transcriptomic context. Panel **(A)** shows PCA/sample context for the curated reference (n = 9) versus severe (n = 6) mouse cochlear contrast. Panel **(B)** provides gene-level transparency; nominal P < 0.05 genes/probes are highlighted, but no gene or probe reached FDR q < 0.05. Panel **(C)** shows the two primary FDR-supported Hallmark pathway anchors, inflammatory response and IL6/JAK/STAT3 signaling. Panel **(D)** shows secondary exploratory interferon and antigen-processing context. Positive NES indicates enrichment in severe mouse cochlea relative to reference. These mouse bulk data provide tissue-context information only and do not validate the human clinical flow-cytometry findings. Complete Hallmark, Reactome, and prespecified immune-focused enrichment results are provided in Supplementary Data S1.

Figure 4 and Table 3 summarize GSE49543 mouse cochlear bulk transcriptomic context. PCA provides sample-level context for the curated reference versus severe contrast. The gene-level transparency panel shows that nominally different genes or probes were present, but no gene or probe reached FDR q < 0.05. This distinction is important: the bulk layer is driven by pathway-level signal rather than by a small set of individual FDR-significant genes.

**TABLE 3:** Public mouse cochlear bulk primary FDR-supported pathway anchors.

Pathway	Gene-set source	NES	P Value	FDR q	Gene-set size	Interpretation
Inflammatory response	MSigDB hallmark	1.6	0.00162	0.0162	182	Primary FDR-supported pathway context
IL6/JAK/STAT3 signaling	MSigDB hallmark	1.54	0.00768	0.0426	86	Primary FDR-supported pathway context

1 NES, normalized enrichment score. FDR, false-discovery rate. Positive NES, indicates enrichment in severe mouse cochlea relative to reference.

2 Public mouse cochlear bulk data provide tissue-context information only.

In the severe versus reference mouse cochlear comparison, no gene or probe reached FDR q < 0.05. Within the prespecified immune-focused enrichment display, inflammatory response (NES 1.60, FDR q = 0.016) and IL6/JAK/STAT3 signaling (NES 1.54, FDR q = 0.043) were the two FDR-supported pathway anchors shown in Figure 4. Interferon and antigen-processing pathways in the same display remained exploratory. Complete Hallmark, Reactome, and immune-focused enrichment results are provided in Supplementary Data S1.

### Public mouse cochlear snRNA-seq broad-label localization context

**FIGURE 5:**
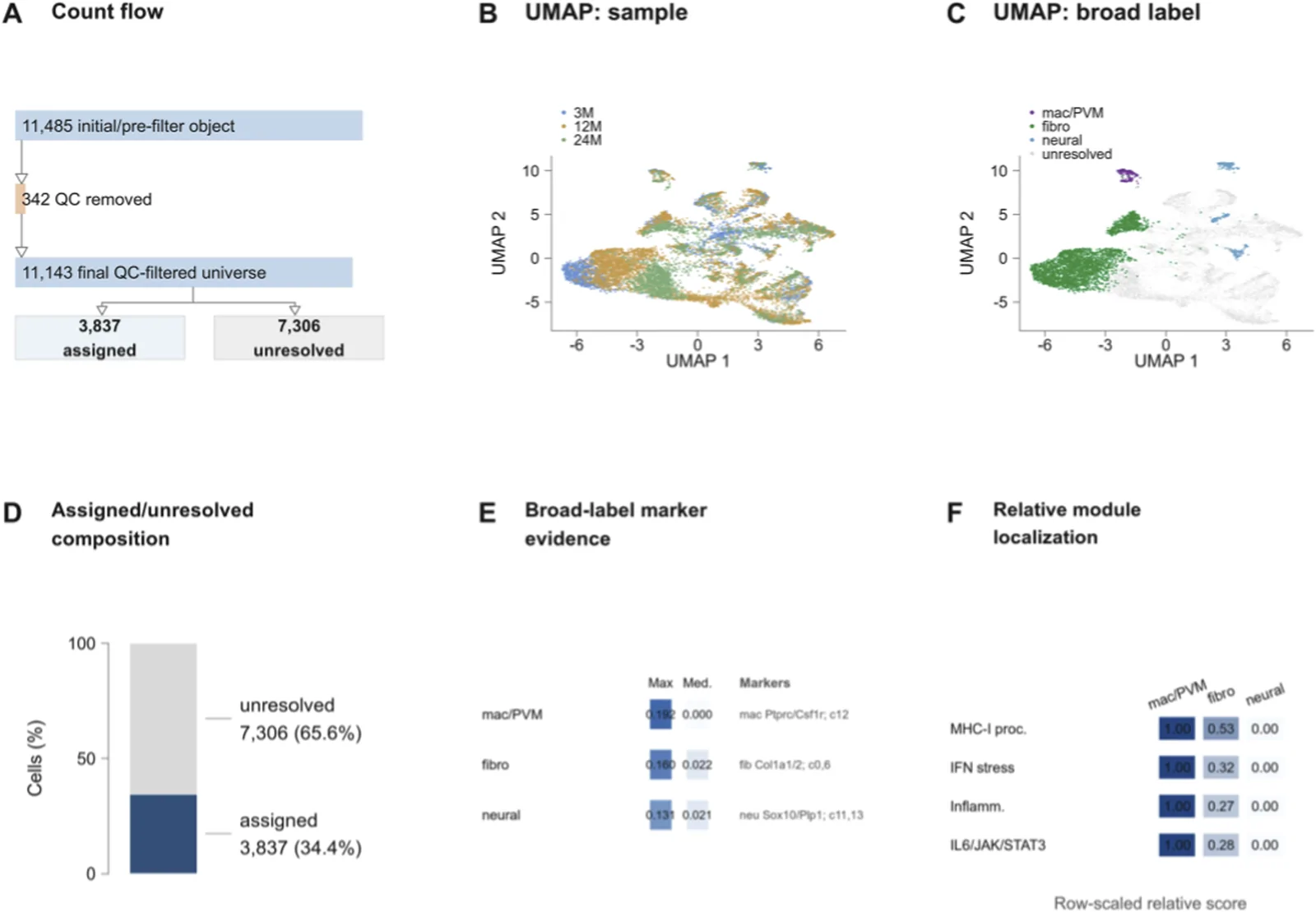
Public mouse cochlear snRNA-seq broad-label localization context summarizes GSE274279 public mouse cochlear snRNA-seq context using exported source tables. The final QC-filtered broad-label/module-scored universe included 11,143 cells, of which 3,837 were assigned to broad labels and 7,306 remained unresolved. Unresolved cells are retained as QC/background and excluded from assigned-label ranking. Marker evidence is derived from exported source data. Module localization uses row-scaled relative scores, where 1.00 indicates the highest-scoring assigned broad label within each module. These outputs do not support abundance-shift inference, do not resolve fine macrophage/perivascular macrophage (mac/PVM) subtypes, and do not provide human cochlear validation. Panel **(A)** shows the count flow of the QC-filtering process. Panel **(B)** displays the UMAP projection colored by sample. Panel **(C)** shows the UMAP projection colored by broad label. Panel **(D)** presents the bar chart of assigned versus unresolved cell composition. Panel **(E)** summarizes the marker evidence for broad labels. Panel **(F)** illustrates the relative module localization scores. IFN, interferon; MHC-I, major histocompatibility complex class I; mac/PVM, macrophage/perivascular macrophage; fibro, fibrocyte/stromal broad label; neural, neural/glial broad label.

Figure 5 summarizes GSE274279 mouse cochlear snRNA-seq context using the final 11,143-cell QC-filtered universe. UMAP panels show sample and broad-label structure. Unresolved cells are displayed as QC/background and are not used for assigned-label ranking. The high unresolved fraction is an important limitation and is retained visually rather than hidden.

Broad-label marker evidence and module-localization panels indicate that selected immune-stress modules ranked highest in the macrophage/perivascular macrophage-like broad label among assigned cells. These values are row-scaled relative scores, not absolute cell-type abundance estimates. Therefore, the snRNA-seq layer is best interpreted as broad-label localization context. It does not demonstrate immune-cell infiltration, expansion of a macrophage population, fine macrophage subtype resolution, or human cochlear confirmation.

The final QC-filtered object contained 11,143 nuclei, of which 3,837 (34.4%) received a broad label and 7,306 (65.6%) remained unresolved. Among assigned broad labels, the selected modules ranked highest in the macrophage/PVM-like category. Detailed sample structure, QC thresholds and counts, marker evidence, module gene sets, localization summaries, and marker-overlap results are provided in Supplementary Data S2.

## Discussion

In this single-center flow-cytometry cohort, SPHL in older adults was associated with a peripheral adaptive immune remodeling profile after adjustment for age and sex. The most consistent participant-level signals involved T-cell/CD8 skew, CD4 differentiation, EM/naive CD4 balance, CD8 activation/checkpoint score, and CD4/CD8 inversion. These findings fit an immune-aging interpretation, but they should not be read as evidence that the measured blood features cause hearing loss or directly reflect cochlear immune status.

The clinical layer is the core of the study. It has several strengths: participant-level flow-cytometry data, a prespecified SPHL versus non-SPHL contrast, age- and sex-adjusted models, and sensitivity analyses that examine age overlap, weighting, and ordered severity. At the same time, the cohort remains modest, the design is cross-sectional, and the available clinical covariates are limited. These constraints are particularly relevant because aging affects both hearing thresholds and peripheral immune composition.

The outcome-correlation analysis helps clarify how the prespecified immune measures relate to one another. The strong correlation between CD4 differentiation and EM/naive CD4 is consistent with their shared representation of CD4 T-cell differentiation state, whereas the remaining correlations were smaller or absent. The outcomes should therefore be interpreted as partially overlapping immune dimensions rather than as six independent biomarkers.

The consistency of effect directions across alternative age specifications, overlap weighting, and leave-one-out refitting reduces concern that the continuous-outcome results depended on a single model specification or observation. The wide bootstrap interval for CD4/CD8 inversion nevertheless reflects the limited number of inversion events and supports cautious interpretation of its effect size.

The observed CD4/CD8 inversion signal is clinically interpretable because it was handled as a binary event and reported on the odds-ratio scale. The event counts also show why caution is needed: SPHL had 10 inversion events among 23 participants, whereas non-SPHL had 4 among 53. The odds ratio was large, but the confidence interval was wide, consistent with a small-event binary model. Replication in an independent cohort would be necessary before treating CD4/CD8 inversion as a stable clinical immune marker in severe hearing loss.

The public mouse cochlear transcriptomic layers add biological context but not validation. GSE49543 showed inflammatory response and IL6/JAK/STAT3 signaling as FDR-supported pathway anchors despite the absence of gene-level FDR-significant findings. This pattern is compatible with pathway-level inflammatory activity in mouse cochlea, but it does not establish that the same pathways are active in human cochlea from the clinical cohort. GSE274279 further suggested that selected immune-stress modules ranked highest in a broad macrophage/perivascular macrophage-like label among assigned cells, a cautious interpretation consistent with prior reports that cochlear macrophage and perivascular macrophage-like populations can participate in cochlear stress and barrier biology, but the high unresolved fraction and broad-label annotation restrict the interpretation (Hirose et al., 2005; Zhang et al., 2012; Yang et al., 2015; Frye et al., 2017).

Several limitations shape the conclusions. First, the study is single-center and includes 76 participants, with 23 in the SPHL group. Second, age imbalance and residual immune-aging confounding cannot be excluded even after adjustment and age-overlap sensitivity analysis. Our study lacked a young healthy control group. However, by focusing on a cohort of older adults and comparing those with severe hearing loss to their peers with normal or + mild-to-moderate loss, our study highlights the immune alterations specifically associated with the severity of hearing loss within an aged population. Third, individual audiometric thresholds, frequency-specific thresholds, comorbidities, exposure history, medication history, and inflammatory or infection markers were unavailable in the clinical analysis dataset. Fourth, public mouse cochlear datasets differ in species, sample source, platform, and biological setting from the human peripheral blood cohort. Finally, the snRNA-seq analysis supports broad-label localization only and does not quantify cell abundance shifts.

The results suggest a focused next step rather than a finished mechanistic model. Future work should test the blood immune phenotype in larger cohorts with individual PTA values, frequency-specific thresholds, key comorbidities, medication and exposurn in an external clinical cohort and orthogonal tissue or experimental evidence would be needed to determine whether the peripheral immune phenotype is a marker, mediator, consequence, or correlate of severe hearing loss in older adults.

## Conclusion

In summary, SPHL in this older adult cohort was associated with peripheral adaptive immune remodeling, and public mouse cochlear datasets provide contextual support, but do not validate the human findings. The study is best interpreted as a conservative clinical association analysis with tissue-contextual support, not as proof of causality or human cochlear immune mechanism.

## Data Availability

Public mouse datasets are available from GEO under GSE49543 and GSE274279. Derived tables and figure source data are provided with the submission materials. Access to clinical source data should follow institutional and ethics constraints.
