# Supplementary material for: Peripheral adaptive immune remodeling associated with severe hearing loss in older adults: a single-center flow-cytometry study with mouse cochlear transcriptomic context
Source: Front Aging. 2026 Sep 3;7:1925166. doi: 10.3389/fragi.2026.1925166 (PMC13582394; doi:10.3389/fragi.2026.1925166)
Supplement: Supplementary file 1 [file Supplementaryfile1.docx]

Supplementary Material

# Supplementary Tables

| **Supplementary Table S1. Flow Cytometry Antibody Panel and Reagent Information** | | | | |
| --- | --- | --- | --- | --- |
| **Marker** | **Fluorochrome** | **Clone** | **Vendor** | **Catalog number** |
| CD3 | PerCP-Cy5.5 | UCHT1 | BD Biosciences | 560835 |
| CD3 | APC-H7 | SK7 (also known as Leu-4) | BD Biosciences | 560176 |
| CD4 | APC-H7 | RPA-T4 | BD Biosciences | 560158 |
| CD56 | APC | B159 | BD Biosciences | 555518 |
| CD8 | BV510 | SK1 | BD Biosciences | 563919 |
| CD94 | PerCP-Cy5.5 | HP-3D9 | BD Biosciences | 562361 |
| CD28 | PE-Cy7 | CD28.2 | BD Biosciences | 560684 |
| NKG2D (CD314) | PE-Cy7 | 1D11 | BD Biosciences | 562365 |
| CD127 | BV421 | HIL-7R-M21 | BD Biosciences | 562436 |
| NKp30 (CD337) | BV421 | p30-15 | BD Biosciences | 563385 |
| CCR7 (CD197) | AF647 | 150503 | BD Biosciences | 560816 |
| NKp46 (CD335) | BV510 | 9E2/NKp46 (also known as 9-E2) | BD Biosciences | 564064 |
| CD25 | PE | M-A251 | BD Biosciences | 555432 |
| NKB1 | PE | DX9 | BD Biosciences | 555967 |
| CD45RA | FITC | HI100 | BD Biosciences | 555488 |
| CD3 | PerCP-Cy5.5 | UCHT1 | BD Biosciences | 560835 |
| CD28 | PE-Cy7 | CD28.2 | BD Biosciences | 560684 |
| CD57 | BV421 | QA17A04 | BioLegend | 393326 |
| CD38 | BV510 | HIT2 | BD Biosciences | 563251 |
| PD-1 (CD279) | AF647 | EH12.1 (also known as EH12) | BD Biosciences | 560838 |
| CD8 | FITC | HIT8a | BD Biosciences | 555634 |
| HLA-DR | PE | G46-6 | BD Biosciences | 555812 |
| CD3 | APC-H7 | SK7 (also known as Leu-4) | BD Biosciences | 560176 |
| TCRγδ | BV421 | 11F2 | BD Biosciences | 744870 |
| PD-1 (CD279) | BB515 | EH12.1 (also known as EH12) | BD Biosciences | 564494 |
| NKp46 (CD335) | BV510 | 9E2/NKp46 (also known as 9-E2) | BD Biosciences | 564064 |
| NKp30 (CD337) | AF647 | p30-15 | BD Biosciences | 558408 |
| NKG2D (CD314) | PE-Cy7 | 1D11 | BD Biosciences | 562365 |
| TCR Vδ2 | PE | B6 | BD Biosciences | 555739 |
| TCR Vδ1 | PerCP-Vio700 | REA173 | Miltenyi Biotec | 130-120-441 |
| CD45 | APC-H7 | 2D1 | BD Biosciences | 560178 |
| CD19 | PerCP-Cy5.5 | HIB19 | BD Biosciences | 561295 |
| CD27 | BV421 | M-T271 | BD Biosciences | 562513 |
| IgD | BB515 | IA6-2 | BD Biosciences | 565243 |
| IgM | BV510 | G20-127 | BD Biosciences | 563113 |
| CD38 | APC | HIT2 | BD Biosciences | 555462 |
| CD24 | PE | ML5 | BD Biosciences | 555428 |
| CD21 | PE-Cy7 | B-ly4 | BD Biosciences | 561374 |
| CD3 | APC-H7 | SK7 (also known as Leu-4) | BD Biosciences | 560176 |
| CD4 | PE-Cy7 | SK3 (also known as Leu-3a) | BD Biosciences | 557852 |
| CD8 | PerCP-Cy5.5 | SK1 | BD Biosciences | 565310 |
| CD183(CXCR3) | AF488 | 1C6/CXCR3 | BD Biosciences | 558047 |
| CD196(CCR6) | BV510 | 11A9 | BD Biosciences | 563241 |
| CD185(CXCR5) | AF647 | RF8B2 | BD Biosciences | 558113 |
| CD194 | BV421 | 1G1 | BD Biosciences | 562579 |
| PD-1 (CD279) | PE | MIH4 | BD Biosciences | 557946 |

| **Supplementary Table S2. Gating Strategies and Definitions for Immune Cell Subpopulations** | | | | | |
| --- | --- | --- | --- | --- | --- |
| **Population** | **Parent gate** | **Denominator** | **Marker definition** | **Related score/table** | **S1 panel** |
| Lymphocytes | FSC/SSC lymphocyte gate | Acquired events | FSC/SSC lymphocyte region | Gating hierarchy | S1A A1; S1B B1 |
| CD3+ T cells | Lymphocytes | Lymphocytes | CD3+ | T-cell and CD4/CD8 gating | S1A A2; S1B B2 |
| CD4+ T cells | CD3+ T cells | CD3+ T cells | CD3+CD4+ branch | CD4 branch definitions | S1A A3; S1B B3-B4 |
| CD8+ T cells | CD3+ T cells | CD3+ T cells | CD3+CD8+ branch | CD8 branch definitions | S1A A3; S1B B3-B5 |
| CD4 naive-memory subsets | CD3+CD4+ T cells | CD3+CD4+ T cells | CCR7/CD45RA-defined subsets | Table S3: CD4 differentiation; EM/naive CD4 | S1B B6 |
| CD8 naive-memory subsets | CD3+CD8+ T cells | CD3+CD8+ T cells | CCR7/CD45RA-defined subsets | CD8 differentiation context | S1B B7 |
| Treg-like cells | CD3+CD4+ T cells | CD3+CD4+ T cells | CD25+CD127low/− | Treg-like gating context | S1A A7; S1C C1 |
| CD8 activation/checkpoint subsets | CD3+CD8+ T cells | CD3+CD8+ T cells | HLA-DR/CD38 and PD-1 subsets as applicable | Table S3: CD8 activation/checkpoint | S1A A8; S1B B8-B9 |
| CD4/CD8 ratio and inversion | CD3+ T cells | CD3+ T cells | Ratio of CD4+ to CD8+ T-cell branches; inversion event is ratio <1 | Table S3: CD4/CD8 inversion | S1A A3; S1B B3 |
| γδT cells | CD3+ T cells | CD3+ T cells | TCRγδ+ or gamma-delta gate as shown | Gamma-delta T-cell gating context | S1A A6; S1C C7 |
| Vδ1/Vδ2 γδT subsets | γδT cells | γδT cells | Vδ1/Vδ2 gate as shown | Gamma-delta T-cell gating context | S1C C8 |
| NK cells | FSC/SSC lymphocyte gate | Lymphocytes | CD3−CD56+ NK-cell parent gate | NK-cell gating context | S1A A4; S1C C2 |
| NK bright/dim subsets | CD3−CD56+ NK cells | CD3−CD56+ NK cells | CD56 intensity-defined bright/dim subsets | NK subset context | S1C C3 |
| NKp30+ NK cells | CD3−CD56+ NK cells | CD3−CD56+ NK cells | NKp30-positive NK-cell subset | Table S3: NK NCR | S1A A5; S1C C4 |
| NKp46+ NK cells | CD3−CD56+ NK cells | CD3−CD56+ NK cells | NKp46-positive NK-cell subset | Table S3: NK NCR | S1C C5 |
| NKG2D+ NK cells | CD3−CD56+ NK cells | CD3−CD56+ NK cells | NKG2D-positive NK-cell subset | NK receptor context | S1C C6 |
| CD94+ NK cells | CD3−CD56+ NK cells | CD3−CD56+ NK cells | CD94-positive NK-cell subset | NK receptor context | Not shown in representative S1; defined in assay panel |
| Tfh/Th/Tc polarization subsets | CD3+CD4+ or CD3+CD8+ T cells | Relevant CD4 or CD8 branch | CXCR5, CXCR3, CCR4, CCR6, and PD-1 combinations as applicable | Assay panel definitions | Not shown in representative S1; defined in assay panel |
| B cells and B-cell subsets | CD45+ lymphocytes | CD45+ lymphocytes | CD19+ B cells with CD27, IgD, IgM, CD21, CD38, and CD24 combinations as applicable | Assay panel definitions | Not shown in representative S1; defined in assay panel |

| **Supplementary Table S3. Calculation Methods for Composite Immune Feature Scores** | | | | |
| --- | --- | --- | --- | --- |
| **Score** | **Components** | **Parent population** | **Transformation** | **Direction** |
| T-cell/CD8 skew | CD8 T-cell proportion; CD4 T-cell proportion; CD4/CD8 ratio | CD3+ T cells | Standardized component score | Higher values indicate CD8/T-cell skew |
| CD4 differentiation | Effector-memory CD4; terminally differentiated CD4; naive CD4 | CD3+CD4+ T cells | Standardized component score | Higher values indicate a more differentiated CD4 profile |
| EM/naive CD4 | Effector-memory CD4; naive CD4 | CD3+CD4+ T cells | Log ratio | Higher values indicate higher effector-memory CD4 relative to naive CD4 |
| CD8 activation/checkpoint | CD8 HLA-DR; CD8 HLA-DR+CD38+; CD8 PD-1 | CD3+CD8+ T cells | Standardized component score | Higher values indicate higher activation/checkpoint marker-defined score |
| NK NCR | NKp30+ NK cells; NKp46+ NK cells | CD3−CD56+ NK cells | Standardized component score | Higher values indicate higher NK natural-cytotoxicity receptor marker-defined score |
| CD4/CD8 inversion | CD4/CD8 ratio | CD3+ T cells | Binary threshold | Event defined as CD4/CD8 ratio <1 |
| Footnote: Composite scores summarize the standardized component features listed in this table, with directions oriented as shown. | | | | |

**Supplementary Table S4. Pairwise correlations among clinical immune outcomes**

| **variable_1** | **variable_2** | **spearman_rho** | **pairwise_n** | **two_sided_p_value** | **bh_fdr_q_value** | **includes_binary_inversion** |
| --- | --- | --- | --- | --- | --- | --- |
| Tcell_CD8_skew_score | CD4_differentiation_score | 0.4501 | 76 | 0.0000 | 0.0002 | no |
| Tcell_CD8_skew_score | log_EMCD4_NaiveCD4 | 0.4962 | 76 | 0.0000 | 0.0000 | no |
| Tcell_CD8_skew_score | CD8_activation_checkpoint_score | -0.4269 | 76 | 0.0001 | 0.0003 | no |
| Tcell_CD8_skew_score | NK_NCR_score | 0.0978 | 76 | 0.4006 | 0.4816 | no |
| Tcell_CD8_skew_score | CD4_CD8_inversion | 0.6715 | 76 | 0.0000 | 0.0000 | yes_descriptive_only |
| CD4_differentiation_score | log_EMCD4_NaiveCD4 | 0.8804 | 76 | 0.0000 | 0.0000 | no |
| CD4_differentiation_score | CD8_activation_checkpoint_score | -0.1961 | 76 | 0.0896 | 0.1344 | no |
| CD4_differentiation_score | NK_NCR_score | 0.0533 | 76 | 0.6472 | 0.6472 | no |
| CD4_differentiation_score | CD4_CD8_inversion | 0.2104 | 76 | 0.0681 | 0.1135 | yes_descriptive_only |
| log_EMCD4_NaiveCD4 | CD8_activation_checkpoint_score | -0.2232 | 76 | 0.0527 | 0.0987 | no |
| log_EMCD4_NaiveCD4 | NK_NCR_score | 0.0997 | 76 | 0.3916 | 0.4816 | no |
| log_EMCD4_NaiveCD4 | CD4_CD8_inversion | 0.2816 | 76 | 0.0137 | 0.0294 | yes_descriptive_only |
| CD8_activation_checkpoint_score | NK_NCR_score | 0.0597 | 76 | 0.6082 | 0.6472 | 0.0111 |
| CD8_activation_checkpoint_score | CD4_CD8_inversion | -0.4425 | 76 | 0.0001 | 0.0002 | yes_descriptive_only |
| NK_NCR_score | CD4_CD8_inversion | -0.0944 | 76 | 0.4174 | 0.4816 | yes_descriptive_only |

**Supplementary Table S5: Mouse Cochlear Transcriptome Data Analysis**

| **Supplementary Table S5A. Sample Metadata and Grouping Information** | | | | |
| --- | --- | --- | --- | --- |
| **Sample ID** | **Analysis include** | **Severity group** | **Primary comparison group** | **Trend order** |
| GSM1201591 | TRUE | mild_presbycusis | reference | 1 |
| GSM1201592 | TRUE | mild_presbycusis | reference | 1 |
| GSM1201593 | TRUE | mild_presbycusis | reference | 1 |
| GSM1201594 | TRUE | mild_presbycusis | reference | 1 |
| GSM1201595 | TRUE | mild_presbycusis | reference | 1 |
| GSM1201596 | TRUE | mild_presbycusis | reference | 1 |
| GSM1201597 | TRUE | mild_presbycusis | reference | 1 |
| GSM1201598 | TRUE | mild_presbycusis | reference | 1 |
| GSM1201599 | TRUE | mild_presbycusis | reference | 1 |
| GSM1201600 | TRUE | severe_presbycusis | severe | 2 |
| GSM1201601 | TRUE | severe_presbycusis | severe | 2 |
| GSM1201602 | TRUE | severe_presbycusis | severe | 2 |
| GSM1201603 | TRUE | severe_presbycusis | severe | 2 |
| GSM1201604 | TRUE | severe_presbycusis | severe | 2 |
| GSM1201605 | TRUE | severe_presbycusis | severe | 2 |

| **Supplementary Table S5B. Analysis of Core Significantly Enriched Pathways in the Mouse Cochlea** | | | | | | |
| --- | --- | --- | --- | --- | --- | --- |
| **Pathway** | **Collection** | **NES** | **P value** | **FDR q** | **Size** | **Tier** |
| Inflammatory response | hallmark | 1.603 | 0.0016 | 0.0162 | 182 | Primary FDR-supported |
| IL6/JAK/STAT3 signaling | hallmark | 1.536 | 0.0077 | 0.0426 | 86 | Primary FDR-supported |
| Footnote: Positive NES indicates enrichment in severe mouse cochlea relative to reference. Public mouse cochlear bulk data provide tissue-context information only. | | | | | | |

| **Supplementary Table S5C. Exploratory Pathway Enrichment Analysis in the Mouse Cochlea** | | | | | | |
| --- | --- | --- | --- | --- | --- | --- |
| **Pathway** | **Collection** | **NES** | **P value** | **FDR q** | **Size** | **Tier** |
| Interferon alpha response | hallmark | 1.446 | 0.0205 | 0.079 | 86 | Exploratory context |
| Interferon gamma response | hallmark | 1.332 | 0.0381 | 0.1359 | 179 | Exploratory context |
| DDX58/IFIH1 mediated induction of interferon alpha/beta | reactome | -1.68 | 0.0059 | 0.1135 | 33 | Exploratory context |
| Antigen processing/proteasome degradation | reactome | -1.377 | 0.0328 | 0.2628 | 69 | Exploratory context |
| Class I MHC-I mediated antigen processing presentation | reactome | -1.262 | 0.0487 | 0.3323 | 118 | Exploratory context |
| Footnote: Positive NES indicates enrichment in severe mouse cochlea relative to reference. Public mouse cochlear bulk data provide tissue-context information only. | | | | | | |

| **Supplementary Table S5D. Summary of Differential Gene Expression Analysis Results** | |
| --- | --- |
| **Summary item** | **Value** |
| Gene-level panel | Shown for transparency; no gene/probe reached FDR q<0.05. |
| Total genes/probes tested | 22690 |
| Nominal P<0.05 genes/probes | 775 |
| FDR q<0.05 genes/probes | 0 |
| Minimum FDR q | 0.2165 |
| Footnote: Gene/probe counts are from the exported Figure 4 source-data workbook. Top nominal genes are not labeled in the main Figure 4 panel. | |

**Supplementary Table S6: Single-Cell RNA Sequencing Data Analysis**

| **Supplementary Table S6A: Statistics of the Single-Cell Data Quality Control Pipeline** | |
| --- | --- |
| **Item** | **Count** |
| Initial/pre-filter QC object | 11485 |
| QC removed | 342 |
| Final QC-filtered universe | 11143 |
| Assigned broad labels | 3837 |
| Unresolved | 7306 |

| **Supplementary Table S6B: Initial Quality Control Metrics for Each Sample** | | | | |
| --- | --- | --- | --- | --- |
| **Sample** | **Cells** | **Median nFeature_RNA** | **Median nCount_RNA** | **Median percent.mt** |
| 3M_Cochlea | 2936 | 1362 | 2404 | 0.473379 |
| 12M_Cochlea | 5581 | 1500 | 2611 | 0.698786 |
| 24M_Cochlea | 2968 | 1043.5 | 1818.5 | 0.039338 |
| Footnote: Counts correspond to the initial/pre-filter QC object; 2,936 + 5,581 + 2,968 = 11,485. | | | | |

| **Supplementary Table S6C: Distribution of Post-QC Cells Across Major Cell Types** | | | |
| --- | --- | --- | --- |
| **Sample** | **Cluster** | **Broad label** | **Cells** |
| 12M_Cochlea | c0 | fibro/stromal | 1670 |
| 12M_Cochlea | c6 | fibro/stromal | 272 |
| 12M_Cochlea | c12 | mac/PVM-like | 75 |
| 12M_Cochlea | c11 | neural/glial | 119 |
| 12M_Cochlea | c13 | neural/glial | 15 |
| 12M_Cochlea | c1 | unresolved | 723 |
| 12M_Cochlea | c2 | unresolved | 407 |
| 12M_Cochlea | c3 | unresolved | 386 |
| 12M_Cochlea | c4 | unresolved | 361 |
| 12M_Cochlea | c5 | unresolved | 441 |
| 12M_Cochlea | c7 | unresolved | 379 |
| 12M_Cochlea | c8 | unresolved | 181 |
| 12M_Cochlea | c9 | unresolved | 231 |
| 12M_Cochlea | c10 | unresolved | 154 |
| 24M_Cochlea | c0 | fibro/stromal | 64 |
| 24M_Cochlea | c6 | fibro/stromal | 238 |
| 24M_Cochlea | c12 | mac/PVM-like | 43 |
| 24M_Cochlea | c11 | neural/glial | 18 |
| 24M_Cochlea | c13 | neural/glial | 34 |
| 24M_Cochlea | c1 | unresolved | 1087 |
| 24M_Cochlea | c2 | unresolved | 532 |
| 24M_Cochlea | c3 | unresolved | 206 |
| 24M_Cochlea | c4 | unresolved | 147 |
| 24M_Cochlea | c5 | unresolved | 105 |
| 24M_Cochlea | c7 | unresolved | 125 |
| 24M_Cochlea | c8 | unresolved | 198 |
| 24M_Cochlea | c9 | unresolved | 33 |
| 24M_Cochlea | c10 | unresolved | 54 |
| 3M_Cochlea | c0 | fibro/stromal | 973 |
| 3M_Cochlea | c6 | fibro/stromal | 82 |
| 3M_Cochlea | c12 | mac/PVM-like | 61 |
| 3M_Cochlea | c11 | neural/glial | 77 |
| 3M_Cochlea | c13 | neural/glial | 96 |
| 3M_Cochlea | c1 | unresolved | 38 |
| 3M_Cochlea | c2 | unresolved | 503 |
| 3M_Cochlea | c3 | unresolved | 121 |
| 3M_Cochlea | c4 | unresolved | 197 |
| 3M_Cochlea | c5 | unresolved | 126 |
| 3M_Cochlea | c7 | unresolved | 78 |
| 3M_Cochlea | c8 | unresolved | 174 |
| 3M_Cochlea | c9 | unresolved | 153 |
| 3M_Cochlea | c10 | unresolved | 166 |

| **Supplementary Table S6D: Marker Gene Evidence for Major Cell Type Annotations** | | | | |
| --- | --- | --- | --- | --- |
| **Broad label** | **Marker genes** | **Supported clusters** | **Max evidence score** | **Median evidence score** |
| mac/PVM-like | Ptprc/Csf1r | c12 | 0.1919 | 0.0001 |
| fibro/stromal | Col1a1/2 | c0,6 | 0.1604 | 0.0221 |
| neural/glial | Sox10/Plp1 | c11,13 | 0.1311 | 0.0211 |

| **Supplementary Table S6E: Activity Scores of Key Functional Modules Across Different Cell Types** | | | | | | |
| --- | --- | --- | --- | --- | --- | --- |
| **Broad label** | **Cells** | **Module** | **Mean score** | **Median score** | **Row-scaled relative score** | **Rank among assigned labels** |
| mac/PVM-like | 179 | IL6/JAK/STAT3 | 0.0654 | 0.0514 | 1 | 1 |
| fibro/stromal | 3299 | IL6/JAK/STAT3 | 0.0076 | 0.0052 | 0.283 | 2 |
| neural/glial | 359 | IL6/JAK/STAT3 | -0.0153 | -0.0181 | 0 | 3 |
| mac/PVM-like | 179 | Inflammatory response | 0.0637 | 0.0536 | 1 | 1 |
| fibro/stromal | 3299 | Inflammatory response | 0.0109 | 0.0095 | 0.268 | 2 |
| neural/glial | 359 | Inflammatory response | -0.0084 | -0.0087 | 0 | 3 |
| mac/PVM-like | 179 | Interferon-linked stress | 1.4211 | 1.1591 | 1 | 1 |
| fibro/stromal | 3299 | Interferon-linked stress | 0.1568 | -0.0027 | 0.323 | 2 |
| neural/glial | 359 | Interferon-linked stress | -0.4464 | -0.5266 | 0 | 3 |
| mac/PVM-like | 179 | MHC-I antigen processing | 0.595 | 0.4481 | 1 | 1 |
| fibro/stromal | 3299 | MHC-I antigen processing | 0.1352 | 0.1371 | 0.534 | 2 |
| neural/glial | 359 | MHC-I antigen processing | -0.3915 | -0.3316 | 0 | 3 |
| Footnote: Module localization uses row-scaled relative scores; 1.00 indicates the highest-scoring assigned broad label within each module. Unresolved cells are excluded from assigned-label ranking. | | | | | | |

## Supplementary Figures

Supplementary Figure S1. Gating strategy for flow cytometry. A: Overview of the gating hierarchy used to identify major lymphocyte subsets. Lymphocytes were first gated based on FSC/SSC parameters, followed by sequential gating using markers including CD3, CD4/CD8, CD19, and CD56 to identify T cells, CD4+ and CD8+ T cell subsets, B cells, NK cells, and Treg cells. B: Detailed gating for T cells and CD4/CD8 subsets. Following the identification of CD3+ T cells, cells were further analyzed for CD4 and CD8 expression. CD4+ and CD8+ T cells were then stained with CD45RA and CCR7 to differentiate their maturation states. C: Detailed gating for Treg-like cells, NK cells, and NCR-related subsets. The strategy demonstrates the identification of Treg cells (CD25+CD127low) from the CD3+CD4+ T cell population, as well as the analysis of Natural Cytotoxicity Receptor (NCR).

Supplementary Figure S2. Quality control metrics and UMAP visualization of single-cell RNA-seq data. A: Uniform Manifold Approximation and Projection (UMAP) plot visualizing the cellular landscape, with cells colored according to their sample of origin. B: UMAP projection showing distinct cell clusters identified by unsupervised clustering. C-E: Density plots showing the distribution of C the number of unique RNA molecules detected per cell (nCount_RNA), D the number of unique genes detected per cell (nFeature_RNA), and E the percentage of mitochondrial reads (percent.mt). F Scatter plot showing the correlation between the number of RNA molecules (nCount_RNA) and the number of detected genes (nFeature_RNA) per cell.

Supplementary Figure S3. Pairwise correlations among clinical immune outcomes. The lower-triangular heatmap shows the 15 unique pairwise Spearman correlations among the six prespecified clinical immune outcomes; all pairs included 76 participants. Red denotes a positive correlation, blue denotes a negative correlation, and the cell value is Spearman rho. Correlations involving CD4/CD8 inversion are descriptive because inversion is binary. Exact two-sided P values and Benjamini-Hochberg-adjusted q values across the 15 correlations are reported in Supplementary Table S4C.
